# Candidate genes in gastric cancer identified by constructing a weighted gene co-expression network

**DOI:** 10.7717/peerj.4692

**Published:** 2018-05-04

**Authors:** Jian Chen, Xiuwen Wang, Bing Hu, Yifu He, Xiaojun Qian, Wei Wang

**Affiliations:** 1 Department of Chemotherapy, Qilu Hospital, Shandong University, Jinan, Shandong, China; 2 Department of Chemotherapy, Anhui Provincial Hospital, Hefei, Anhui, China

**Keywords:** Gastric cancer, Weighted gene co-expression network analysis, Candidate gene, Histologic grade, Overall survival, Pathologic T stage

## Abstract

**Background:**

Gastric cancer (GC) is one of the most common cancers with high mortality globally. However, the molecular mechanisms of GC are unclear, and the prognosis of GC is poor. Therefore, it is important to explore the underlying mechanisms and screen for novel prognostic markers and treatment targets.

**Methods:**

The genetic and clinical data of GC patients in The Cancer Genome Atlas (TCGA) was analyzed by weighted gene co-expression network analysis (WGCNA). Modules with clinical significance and preservation were distinguished, and gene ontology and pathway enrichment analysis were performed. Hub genes of these modules were validated in the TCGA dataset and another independent dataset from the Gene Expression Omnibus (GEO) database by *t*-test. Furthermore, the significance of these genes was confirmed via survival analysis.

**Results:**

We found a preserved module consisting of 506 genes was associated with clinical traits including pathologic T stage and histologic grade. PDGFRB, COL8A1, EFEMP2, FBN1, EMILIN1, FSTL1 and KIRREL were identified as candidate genes in the module. Their expression levels were correlated with pathologic T stage and histologic grade, also affected overall survival of GC patients.

**Conclusion:**

These candidate genes may be involved in proliferation and differentiation of GC cells. They may serve as novel prognostic markers and treatment targets. Moreover, most of them were first reported in GC and deserved further research.

## Introduction

GC (gastric cancer) is the fifth most frequently diagnosed cancer and the third leading cause of death from cancer worldwide. In the year of 2015, 1,313,000 people were diagnosed and 813,000 people died from GC (Global Burden of Disease Cancer Collaboration et al., 2017). It is often diagnosed at an advanced stage since lack of specific early symptoms and chemotherapy is the main treatment (Hohenberger & Gretschel, 2003). However, the median survival of patients with advanced GC is less than one year (GASTRIC Group et al., 2013). In recent years, anti-HER2 therapy has prolonged the survival of patients whose HER2 amplified or overexpressed, but the rate of HER2-positivity is less than 30% in patients with advanced GC (Bang et al., 2010). Anti-angiogenic therapy is another strategy for the treatment of patients with advanced GC. However, it prolongs the overall survival by less than two months (Fuchs et al., 2014; Wilke et al., 2014). That means a small subset of patients may benefit from these target therapies. Consequently, it is extremely crucial to discover novel candidate genes, which play important roles in tumorigenesis and could be new targets for treatment.

Technological development of microarray and high-throughput sequencing have shed new light on the research of molecular mechanisms and screening for drug targets of tumors. The Cancer Genome Atlas (TCGA), has provided a huge amount of publicly available genomic and clinical data of many cancer types to help researchers around the world to better study and understand the biology and pathology of each cancer. So far, a few molecular mechanisms and characters in various cancers have been uncovered based on it (Tomczak, Czerwińska & Wiznerowicz, 2015). To explore the underlying mechanisms and identify novel prognostic markers and treatment targets of gastric cancer, in this study, weighted gene co-expression network analysis (WGCNA) was performed on RNA sequencing data of GC patients from TCGA, and significant modules and genes were identified. These genes were confirmed in other independent datasets and may act as oncogenes. They are potential prognostic biomarkers or therapeutic targets probably.

## Materials & Methods

### Acquiring and preparing genetic and clinical data

RNA sequencing and clinical data of GC patients were downloaded from TCGA data repository (https://cancergenome.nih.gov/). The gene expression level was measured as fragments per kilobase of transcript per million mapped reads (FPKM). Clinical data contained the pathologic TNM stage, histologic grade, and survival information. Samples with either pathologic stage or histologic grade information incomplete were not included. Raw data of gene expression of GSE15459 and GSE26942 datasets were downloaded from Gene Expression Omnibus (GEO) database (https://www.ncbi.nlm.nih.gov/geo/). The clinical data of the GSE15459 dataset was obtained from a published literature (Ooi et al., 2009). Samples with either pathologic stage or histologic grade information incomplete were excluded. Both datasets obtained from GEO served as validation datasets.

As genes with little variation in expression usually represent noise, the most variant genes were filtered for network construction. Gene variabilities were measured by median absolute deviation (MAD).

### Constructing gene co-expression network

Gene co-expression network was constructed by the WGCNA package (Langfelder & Horvath, 2008) in R. Before co-expression network construction, squared Euclidean distance of each sample was calculated by function adjacency, and whole sample network connectivity according to distance was standardized by function scale. The outlier samples whose connectivity less than −2.5 were excluded (Horvath, 2011). Function pickSoftThreshold was used to calculate scale-free topology fitting indices *R*^2^ corresponding to different soft thresholding powers *β*. The *β* value was chosen as long as *R*^2^ reached 0.8. After that, the gene expression matrix was transformed into an adjacency matrix and a Topological Overlap Matrix (TOM), and then the corresponding dissimilarity of TOM (dissTOM) was calculated. For module detection, hierarchical clustering was used to produce a hierarchical clustering tree (dendrogram) of genes by function hclust based on dissTOM. The Dynamic Tree Cut method was performed for branch cutting to generate modules. During this, a relatively large minimum module size of minClusterSize = 30, and a medium sensitivity (deepSplit = 2) to branch splitting were chosen to avoid generating too many small or large modules. The module eigengene (ME), which can be considered as a representative of the gene expression profiles of a module, is defined as the first principal component of a given module. It was calculated by function moduleEigengenes. Modules would be merged if their correlation of MEs was greater than 0.75, which means they have similar expression profiles. Co-expression networks of modules were visualized by Cytoscape software (v3.4.0) (Shannon et al., 2003).

### Identifying preserved modules associated with clinical traits

The correlation between MEs and clinical traits including pathologic stage and histologic grade was evaluated by Pearson’s correlation tests, and *p* < 0.05 was considered to be significantly correlated.

Module preservation, which is used to evaluate whether a module is robust and reproducible across datasets, was calculated by the modulePreservation function (Langfelder et al., 2011). If preservation statistics Zsummary >10, there is strong evidence that the module is preserved. Preservation statistics medianRank is negatively correlated with module preservation.

### Gene ontology and pathway Enrichment analysis

To explore the potential biological themes and pathways of genes in the modules, the clusterprofiler package (Yu et al., 2012) in R was used to annotate and visualize gene ontology (GO) terms and Kyoto Encyclopedia of Genes and Genomes (KEGG) pathways.

### Screening for candidate genes with clinical significance

Module Membership (MM) of a gene represents the membership of the gene with respect to the module. Highly connected intramodular genes, which tend to have high module membership values, were defined as hub genes. To verify the clinical significance of these hub genes, independent *t*-test was used to analyze the expression difference of these genes in samples with different clinical traits in the TCGA dataset and the GSE15459 dataset respectively. Furthermore, significant genes were confirmed via survival analysis. Overall survival data of the TCGA dataset was analyzed. The R package survival was used to carry out log-rank tests and plot Kaplan–Meier survival curves. Moreover, the online software Kaplan Meier plotter (http://kmplot.com/analysis/index.php?cancer=gastric&p=service) (Lánczky et al., 2016), which was capable to implement log-rank tests based on other independent datasets, was used for the further verification.

## Results

### Gene co-expression network of GC

**Figure 1:**
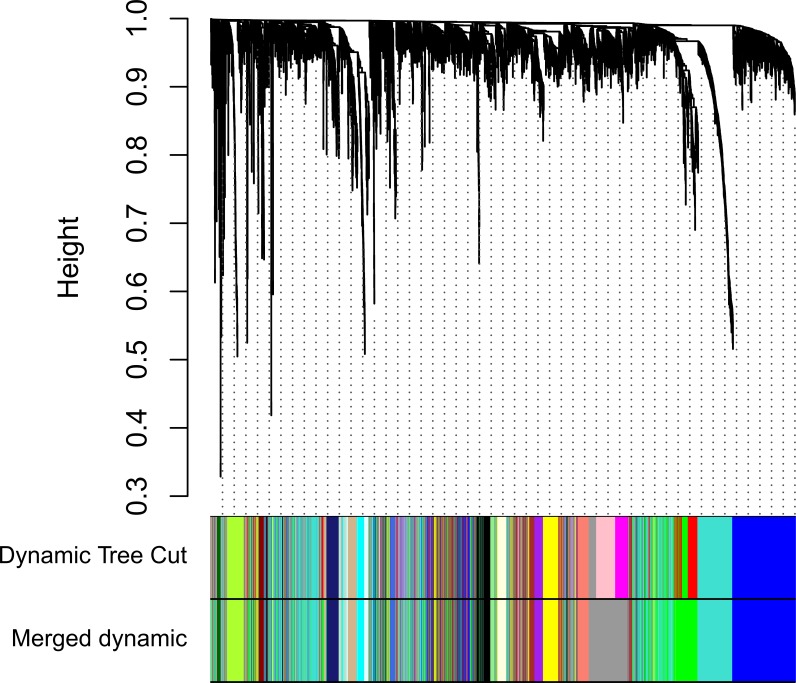
Clustering dendrogram of genes. The hierarchical clustering tree was produced by hierarchical clustering based on dissTOM of genes. Twenty-three modules were identified by Dynamic Tree Cutting method with a medium sensitivity (deepSplit = 2) to branch splitting. Each module was assigned a color as an identifier. Twenty modules were generated after merging according to the correlation of modules. In the colored rowrs below the dendrogram, the two colored rows represent the original modules and merged modules.

Clinical and level-3 RNA sequencing data for 330 gastric adenocarcinoma samples were obtained from TCGA database. For module detection, the 5,000 most variant genes according to MAD value were selected for further analysis from the original over 16,000 protein-coding genes, and two outlier samples were removed according to sample network. When the value of soft thresholding power *β* was 4, the connectivity between genes met a scale-free network distribution (Fig. S1). Twenty-three modules were identified by hierarchical clustering and the Dynamic branch Cutting. Each module was assigned a unique color as an identifier. The ME of each module was calculated (Table S1). Among these modules, three were merged into others because of the similar MEs. Twenty modules were generated finally (Fig. 1, Table S2). The number of genes in modules ranged from 31 to 1,090 (Table S3). The grey module represented a gene set that was not assigned to any of the modules.

### Modules with clinical significance and preservation

**Figure 2:**
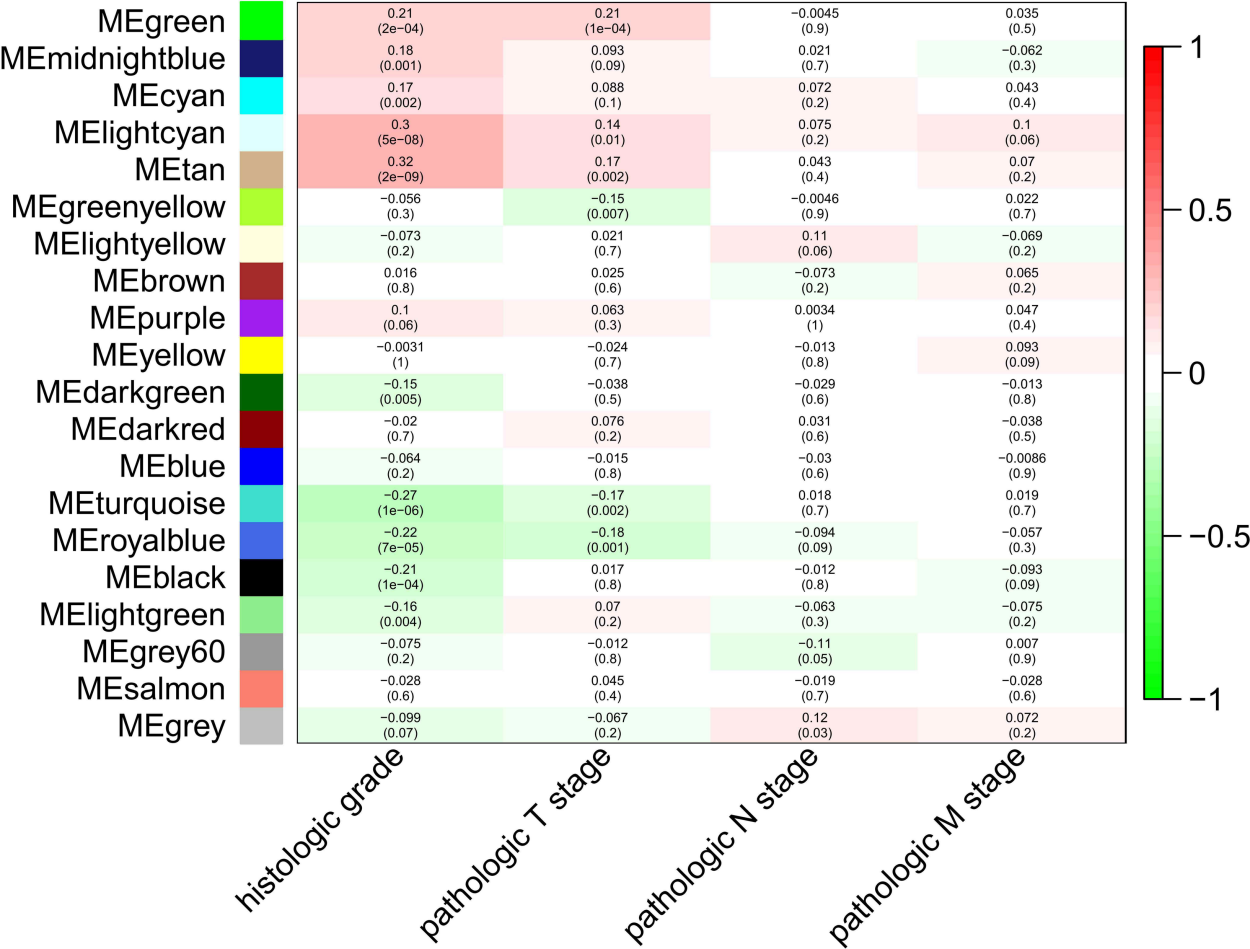
Module-trait associations were evaluated by correlations between MEs and clinical traits. Each row corresponds to a module eigengene, column to a trait. Each cell contains the corresponding correlation (first line) and *p*-value (second line ). The table is color-coded by correlation according to the color legend. Green, midnightblue, cyan, lightcyan and tan modules positively correlated to histologic grade (*p* < 0.05). Darkgreen, turquoise, royalblue, black and lightgreen modules negatively correlated to histologic grade (*p* < 0.05). Green, lightcyan and tan modules positively correlated to pathologic T stage (*p* < 0.05). Greenyellow, turquoise and royalblue modules negatively correlated to pathologic T stage (*p* < 0.05). The grey modules positively correlated to pathologic N stage (*p* < 0.05).

To explore the clinical significance of the module, correlations between MEs and pathologic stage and histologic grade were analyzed. There were five and three modules positively correlated with pathologic T stage and histologic grade, respectively, while another five and three modules negatively correlated with T stage and histologic grade, respectively. There was only one module positively correlated with pathologic N stage, and no module correlated with pathologic M stage (Fig. 2).

**Figure 3:**
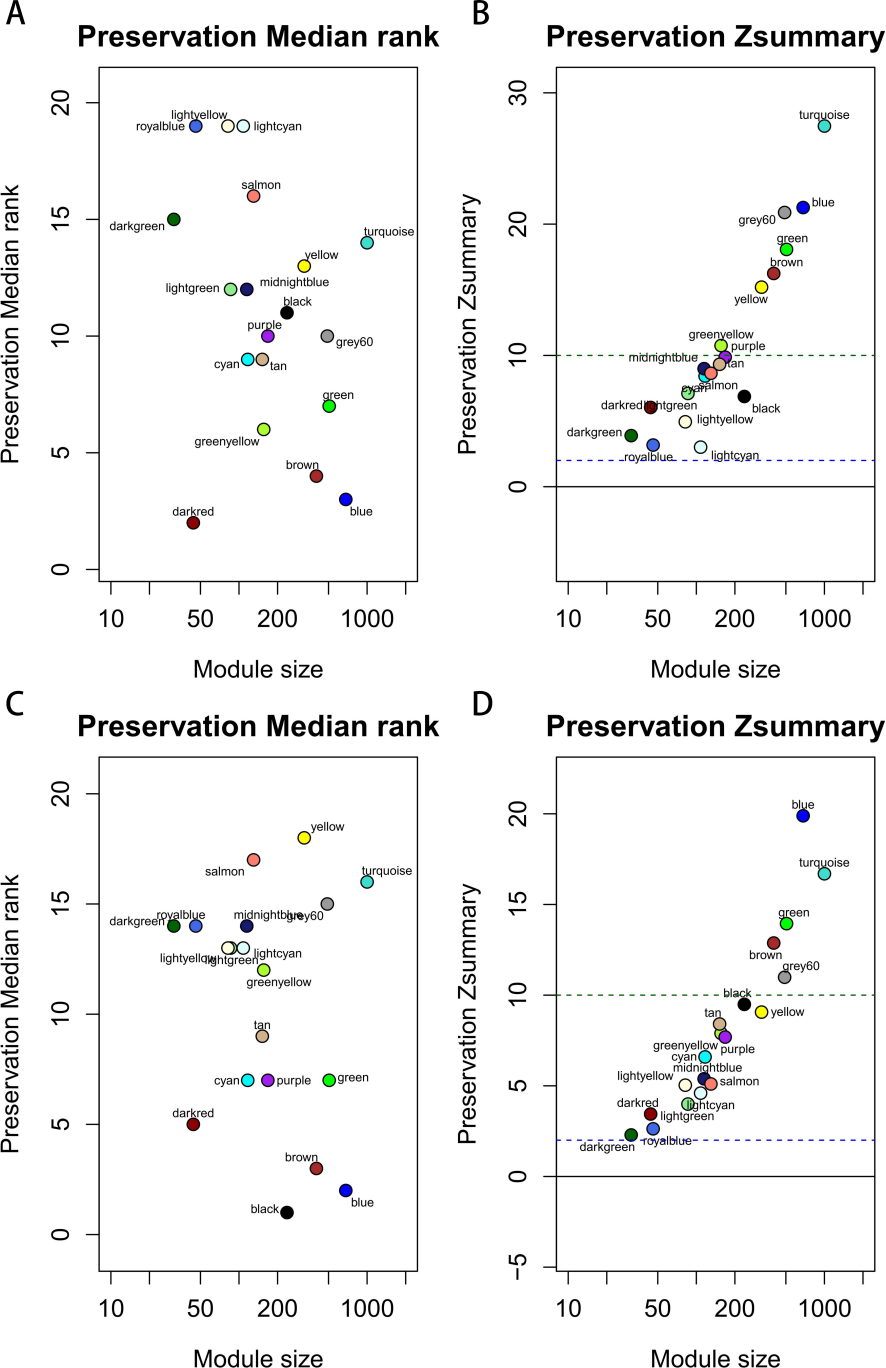
The medianRank and Zsummary statistics of module preservation. Module preservation was evaluated by medianRank and Zsummary statistics which correlated to connectivity and density of networks. If Zsummary >10, there is strong evidence that the module is preserved. The module with lower medianRank tends to exhibit stronger observed preservation than the module with a higher medianRank if both of them are preserved. Compared with the GSE15459 dataset, turquoise, blue, grey60, green, brown, yellow and greenyellow modules with high Zsummary (>10) (A) and low Medianrank statistics (B) were preserved; Compared with the GSE26942 dataset, turquoise, blue, grey60, green and brown modules with high Zsummary (>10) (C) and low Medianrank statistics (D) were preserved.

**Figure 4:**
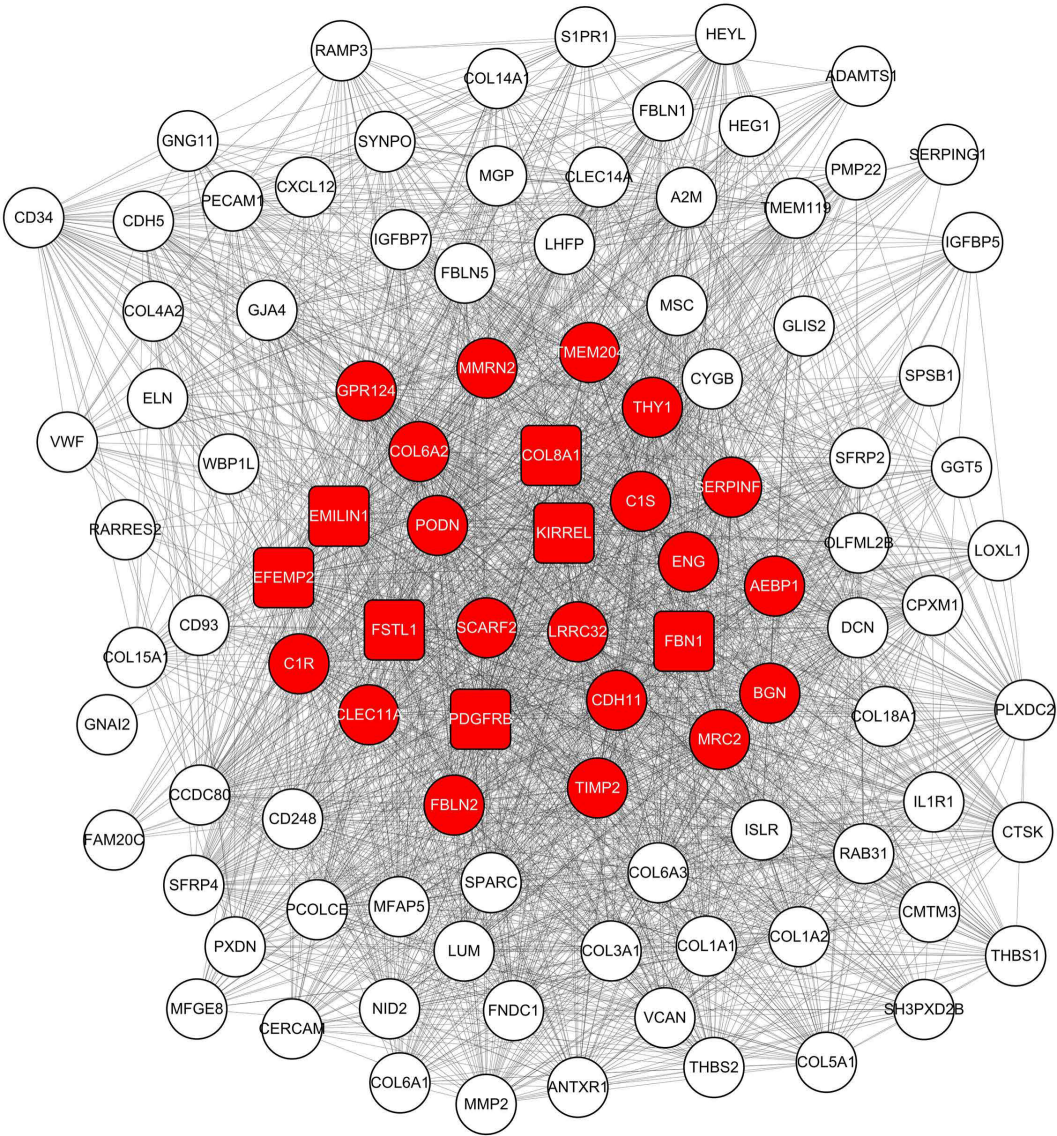
Co-expression network of 100 most connected genes in the green module. Nodes and lines represent genes and correlation between genes. Red nodes are the hub genes of the network.

Before module preservation analysis, six outlier samples in the GSE26942 dataset and five outlier samples in the GSE15459 dataset were excluded according to sample network. When compared with the GSE26942 dataset and GSE15459 dataset, there were five and seven preserved modules respectively, whose Zsummery statistics were greater than 10 and Medianrank statistics were relatively low. Turquoise, blue, grey60, green and brown modules were preserved in both validation datasets (Fig. 3). Among these modules with preservation, according to the previous analysis of clinical significance of modules, green and turquoise modules were correlated with pathologic T stage and histologic grade. Then, the green module was further analyzed while the turquoise module was discarded because of the low MM value of genes in it. Gene co-expression network of the green module was established and visualized by Cytoscape (Fig. 4).

### Enrichment analysis of the green module

**Figure 5:**
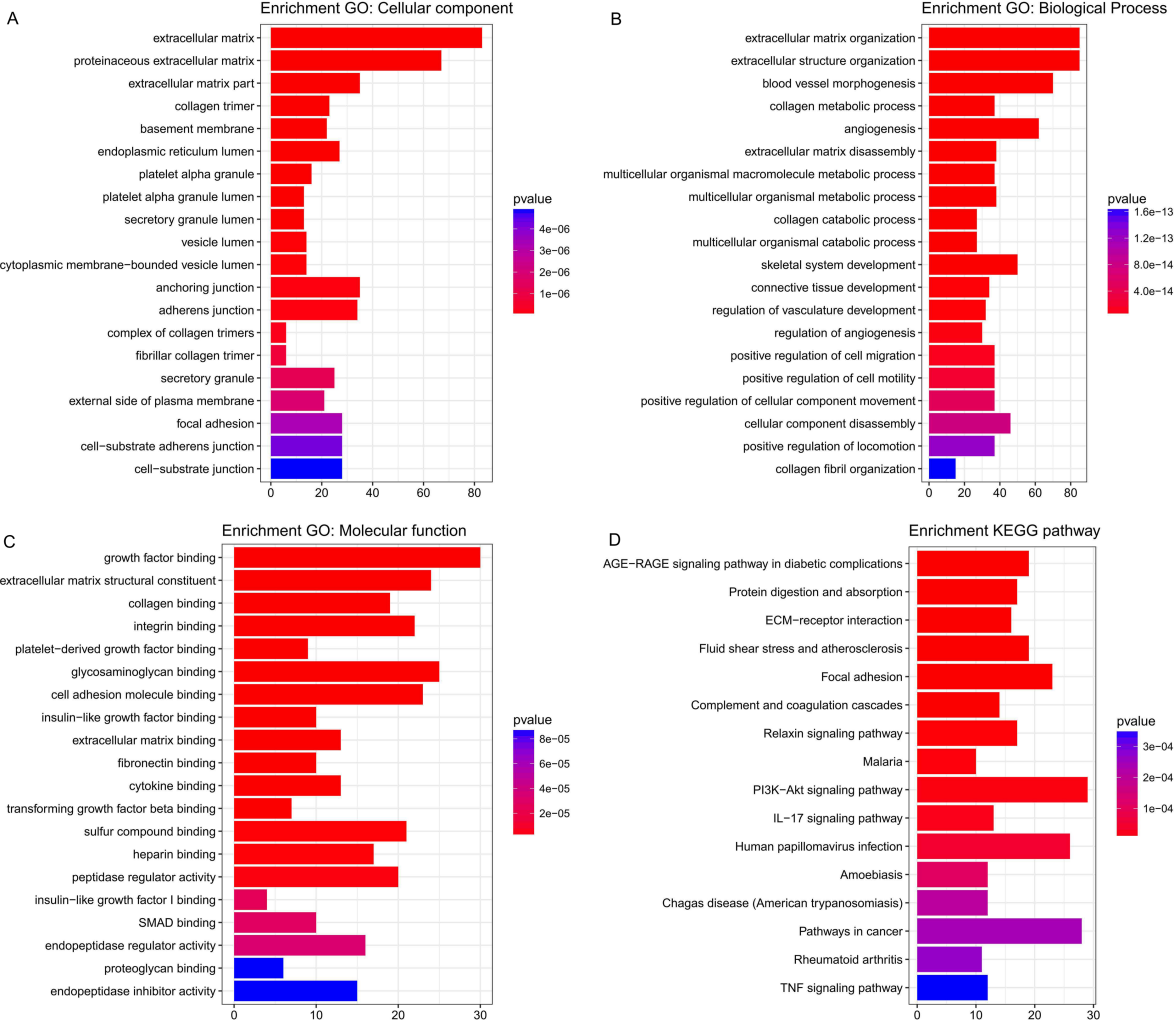
The most significantly enriched GO annotations and pathways of genes in the green module. The length of bars represents the numbers of genes, the color of bars corresponds to *p* value according to legend. (A) Top 20 significantly enriched Cellular component GO annotations; (B) top 20 significantly enriched Biological process GO annotations; (C) top 20 significantly enriched Molecular function GO annotations; (D) top 20 significantly enriched KEGG pathways.

Five hundred and six genes in the green module were mapped to GO database to get their potential functions. The results showed that extracellular matrix, extracellular matrix organization and growth factor binding were the most significant enrichments in cellular component (CC), biological process (BP) and molecular function (MF) groups respectively. In addition, these genes were also involved in angiogenesis, endothelial cell proliferation and insulin-like growth factor binding, which have been proved to be related to cancer (Figs. 5A–5C). KEGG pathway enrichment analysis suggested AGE-RAGE signaling pathway in diabetic complications was the most significant pathway. These genes also participated in ECM-receptor interaction, focal adhesion, PI3K-Akt signaling pathway, pathways in cancer and TNF signaling pathway (Fig. 5D). All of them were cancer-related signal pathways. In addition, the results of enrichment analysis on other modules were shown in supplemental materials (Tables S4– S7).

### Identification and validation of candidate genes

The significance of hub genes with high MM value in a module was consistent with the significance of the module. These genes are also centers of the network and play important roles in the network. Twenty-six genes with MM value greater than 0.8 in the green module were identified as hub genes. The expression levels of hub genes of patients with different pathologic T stages and histologic grades were analyzed by *t*-test. In the TCGA dataset, 24 genes were significantly differentially expressed (*p* < 0.05). In the GSE15459 dataset, eight of these 24 hub genes were also differentially expressed (*p* < 0.05). They were Elastin Microfibril Interfacer 1 (EMILIN1), Collagen Type VIII Alpha 1 Chain (COL8A1), Follistatin Like 1 (FSTL1), EGF Containing Fibulin Like Extracellular Matrix Protein 2 (EFEMP2), Fibrillin 1 (FBN1), Kin of IRRE Like (KIRREL), Platelet Derived Growth Factor Receptor Beta (PDGFRB) and Mannose Receptor C Type 2 (MRC2). All of them were expressed at a low level in T 1 stage or grade 1–2 patients while at a high level in T 2–4 or grade 3 patients in both datasets (Tables 1,  2). These results suggested that they may be associated with proliferation and differentiation of GC cells.

**Figure 6:**
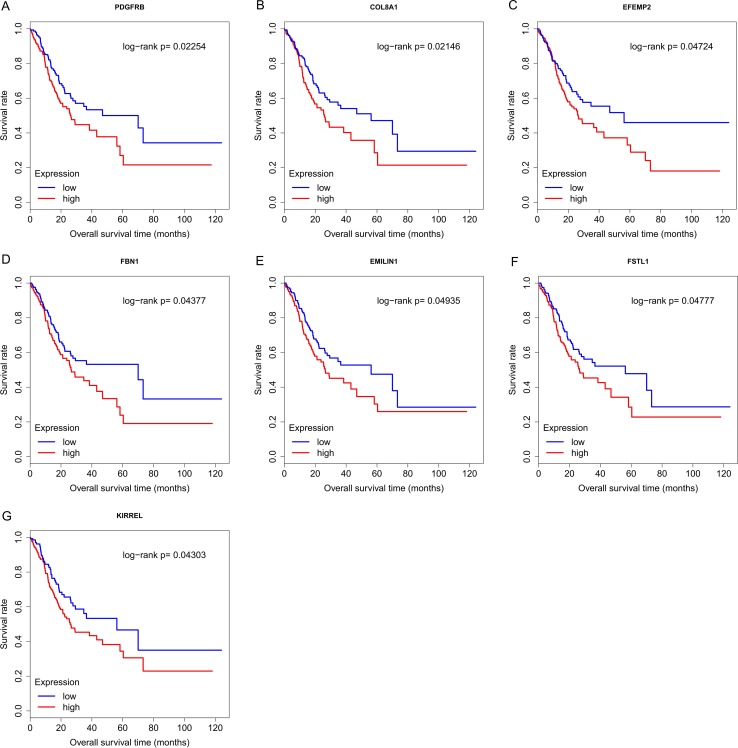
Survival analysis on seven candidate genes based on TCGA dataset. For each gene, the median overall survival of patients in low expression group was superior to that of patients in high expression group (*p* < 0.05). (A) PDGFRB; (B) COL8A1; (C) EFEMP2; (D)FBN1; (E) EMILIN1; (F) FSTL1; (G) KIRREL.

**Figure 7:**
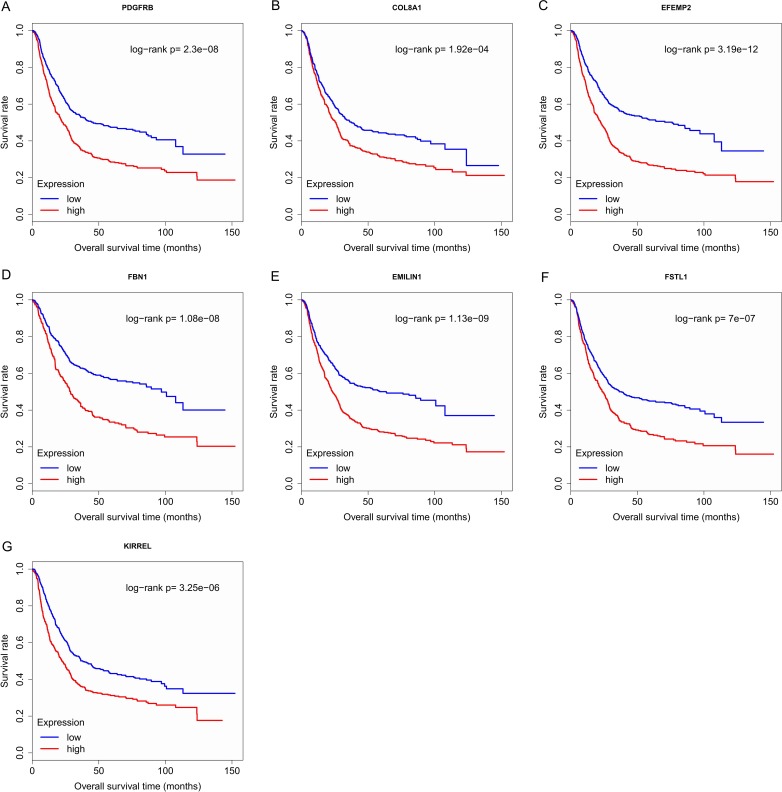
Survival analysis on seven candidate genes based on Kaplan Meier plotter. For each gene, the median overall survival of patients in low expression group was superior to that of patients in high expression group (*p* < 0.05). (A) PDGFRB; (B) COL8A1; (C) EFEMP2; (D) FBN1; (E) EMILIN1; (F) FSTL1; (G) KIRREL.

Finally, the eight hub genes whose expression level correlated to pathologic T stage and histologic grade were verified via survival analysis. The RNA sequencing data and survival information in the TCGA dataset were subjected to survival analysis. Significant different overall survival between the high expression groups and the low expression groups for PDGFRB, COL8A1, EFEMP2, FBN1, EMILIN1, FSTL1 and KIRREL was observed (*p* < 0.05). Their expression levels were associated with overall survival. Furthermore, to confirm these results in other independent datasets, survival analysis was performed through online software Kaplan Meier plotter and consistent positive results were generated (*p* < 0.05). Patients with lower gene expression had longer overall survival and vice versa (Figs. 6, 7). Therefore, the expression levels of PDGFRB, COL8A1, EFEMP2, FBN1, EMILIN1, FSTL1and KIRREL could be prognosticators of survival in GC patients. These genes could be candidate genes of GC for further research.

**Table 1:** Expression levels of genes in different pathologic T stage groups.

Gene	TCGA dataset			GSE15459 dataset		
	Stage T1^*^	Stage T2-4^*^	*p*-Value	Stage T1^*^	Stage T2-4^*^	*p*-Value
PDGFRB	11.27673	28.64472	0.000268	7.650427	8.468733	0.007058
COL8A1	1.405193	8.952865	0.000329	4.607863	5.796504	0.000669
EFEMP2	2.462167	7.409326	6.12E−05	7.862728	8.703876	0.007538
FBN1	2.676911	10.45695	0.000291	7.109844	8.152165	0.015591
MRC2	6.519095	14.13593	0.002959	8.24578	8.609288	0.035221
EMILIN1	7.989158	34.4808	9.30E−05	7.404825	8.254327	0.025756
FSTL1	13.30806	33.73668	0.000217	7.981347	8.575384	0.034181
KIRREL	3.34838	7.0049	0.001232	5.479368	5.832521	0.021507

**Notes.**

* Mean expression level.

**Table 2:** Expression levels of genes in different histologic grade groups.

Gene	TCGA dataset			GSE15459 dataset		
	Grade 1–2^*^	Grade 3^*^	*p*-Value	Grade 1–2^*^	Grade 3^*^	*p*-Value
PDGFRB	23.49514	30.17688	0.002416	8.250134	8.554542	0.020089
COL8A1	6.070836	10.00019	8.11E−05	5.544219	5.87443	0.023504
EFEMP2	5.779241	7.942439	0.000186	8.452001	8.811539	0.009623
FBN1	7.833488	11.33083	0.000521	7.800408	8.313964	0.008226
MRC2	11.80371	14.8516	0.009811	8.447961	8.693392	0.002748
EMILIN1	25.01557	37.77161	6.65E−05	8.017554	8.350455	0.042902
FSTL1	26.17743	36.42849	8.79E−05	8.317364	8.708802	0.003181
KIRREL	5.763699	7.420478	0.001808	5.705279	5.893113	0.007445

**Notes.**

* Mean expression level.

## Discussion

Regulatory network methods are widely used for analyzing gene expression data, especially large datasets. They provide a systematic interpretation of underlying molecular mechanisms and valuable biomarkers associated with disease. Compared with node-based methods, regulatory network methods focus on not only differences but also correlations between gene expression profiles. Therefore, they are more reasonable (Liu, 2016). Many methods to construct gene regulatory network have been developed (Liu, 2015). Liu et al. (2012) employed a method based on differential networks and identified 34 biomarker genes with diagnostic value in gastric cancer. However, this method used a hard thresholding to determine the correlation between genes and did not take into account changes in the strength of the correlation between genes under different conditions.

WGCNA, one of regulatory network methods, is based on power law distribution (scale-free topology) and constructing weighted networks by a soft thresholding. It can detect modules, identify hub genes, and recognize candidate genes or modules relating to external information. It has been proved that WGCNA outperforms many other methods in constructing the global network structure (Allen et al., 2012) and can safely replace mutual information networks based on non-linear gene expression associations (Song, Langfelder & Horvath, 2012) such as ARACNE (Margolin et al., 2006). Therefore, it is a robust method to understand gene expression information and has been widely and successfully applied in various biological contexts (McKinney et al., 2015; Amin et al., 2016; Zhang et al., 2016).

However, to our knowledge, there was only one study researching gene expression profiles of GC by WGCNA (Zhao et al., 2016). This study constructed gene co-expression network, identified modules based on differential expressed genes and performed GO and KEGG pathway enrichment analysis on modules. Finally, genes related to survival were identified by Cox regression analysis. Nevertheless, it did not identify hub genes and analyze the relationship between modules or hub genes and clinical traits. Furthermore, it is not recommended to filter genes by the differential expression for WGCNA because it completely invalidates the scale-free topology assumption and forms a single or a few highly correlated modules.

In our present study, 5,000 most variant genes were filtered by MAD for the reason that genes with little variation are less likely to have high MM value or will not be assigned to a module. We identified modules and candidate genes associated with pathologic T stage and histologic grade of GC. They may play important roles in proliferation and differentiation of gastric adenocarcinoma cells and could be novel targets for the treatment of GC. Also, the expression levels of these candidate genes were associated with overall survival of GC patients, and they could be prognostic biomarkers for GC.

The enrichment analysis of genes in the green module revealed that they could encode protein functioning as growth factor binding, platelet-derived growth factor binding, insulin-like growth factor binding, transforming growth factor beta binding, etc. They were also involved in the MF category and pathways relevant to tumorigenesis and tumor progression such as angiogenesis, PI3K-Akt signaling pathway, pathways in cancer, TNF signaling pathway, etc. These results can explain the correlations between the green module and pathologic T stage and histologic grade.

We defined 26 genes with MM value greater than 0.8 as hub genes, and 24 of them were differentially expressed according to different clinical traits in the TCGA dataset. However, only eight genes were differentially expressed in the GSE15459 dataset. The explanation for this could be the different genetic background of samples in these datasets. Most samples in the TCGA dataset were white or black people while samples in the GSE15459 dataset came from Singapore in Asia. However, the eight genes exhibited stable properties consistent with the green module across datasets.

Among these candidate genes, PDGFRB is the most studied gene related to cancer. Its production is one of the receptors of platelet-derived growth factor (Andrae, Gallini & Betsholtz, 2008), which stimulates proliferation and migration of cancer cells and angiogenesis, and has been utilized as a target for several cancer treatments. It also has been demonstrated that overexpression of PDGFRB is correlated with progression of gastric carcinoma (Guo et al., 2013). This was consistent with the result of our present study and FDGFRB might also be a potential target for GC treatments.

COL8A1, EFEMP2 and FBN1 with activities of oncogenes, have been reported in many studies. The protein encoded by COL8A1 is a component of endothelium of blood vessels. It is necessary for migration and proliferation of vascular smooth muscle cells. Serum concentrations of COL8A1 increases in disease associated with vascular remodeling and forming including cancer (Hansen et al., 2016). EFEMP2 encodes an extracellular matrix protein which is a mutant p53-specific protein partner and contains epidermal growth factor domain. It can enhance oncogenic activity such as neoplastic transformation and proliferation of cancer cells (Gallagher et al., 1999). EFEMP2 is upregulated in gliomas and promotes glioma cell proliferation and invasion (Wang et al., 2015). It is also a promising serum biomarker for colorectal cancer early detection (Yao et al., 2012). The protein encoded by FBN1 is microfibrils of the extracellular matrix. This protein contributes to tissue homeostasis by interactions with growth and differentiation factors, cell–surface integrins and other extracellular matrix protein. FBN1 silencing leads to decreased papillary thyroid carcinoma cell proliferation and enhances apoptosis *in vitro*, up-regulation of FBN1 boosts xenograft tumor formation *in vivo* (Ma et al., 2016). It is overexpressed in testicular germ cell tumors and could be a new marker of germ cell neoplasia *in situ* (Cierna et al., 2016). However, there have been no studies on the correlation between COL8A1, EFEMP2 and FBN1 and GC. Our findings demonstrated that COL8A1, EFEMP2 and FBN1 correlate to pathologic T stage, histologic grade and overall survival in GC patients. They may play important roles in proliferation and differentiation of GC cells. These results in GC were similar to those in other various cancers.

There is controversy about the roles of EMILIN1 and FSTL1 in cancer. EMILIN1 encodes a protein which is responsible for elastogenesis and also regulates the bioavailability of TGF-β. It may suppress proliferation and metastasis of lung cancer cell (Edlund et al., 2012) and is expressed at a lower level in breast cancer than in normal tissue (Rabajdova et al., 2016). However, the opposite results are observed in soft tissue osteosarcoma and ovarian serous tumors (Salani et al., 2007; Rao et al., 2013). FSTL1 encodes an activin-binding protein with similarity to follistatin. It has been proved that FSTL1 activates NFκB and regulates the TGF-β/BMP pathway involved in cell differentiation and subsequent apoptosis (Wu et al., 2015). Knockdown of FSTL1 induces apoptosis of lung cancer cells (Bae et al., 2016). In contrast, FSTL1 shows suppressive effects on ovarian and endometrial carcinogenesis (Chan et al., 2009). Both EMILIN1 and FSTL1 exert contrasting effects in different types of cancer probably because both interact with TGF-β which is a paradigm of duality in cancer (Massagué, 2008). However, there have been no reports on the roles of EMILIN1 and FSTL1 in GC and our study showed for the first time that they may be pro-tumorigenic in GC.

The protein encoded by KIRREL may be involved in glomerular permeability. It can also modulate ERK signaling through interaction with growth factor receptor-bound protein 2 (Harita et al., 2008). So far, there have been no direct researches on the relationship between KIRREL and tumors. However, our study manifested that KIRREL may promote the development of GC. It may be a novel oncogene and worth further study.

## Conclusions

In summary, we identified PDGFRB, COL8A1, EFEMP2, FBN1, EMILIN1, FSTL1 and KIRREL as candidate genes in GC. Most of them were first found to be associated with GC. Their expression levels related to pathologic T stage, histologic grade and overall survival of patients, and this means they may be involved in the development and progression of GC. Thus, the candidate genes we identified can be novel prognostic biomarkers or therapeutic targets of GC and deserve further study. These results, of great clinical significance, will provide new insight into understanding GC and will contribute to personalized therapy.

##  Supplemental Information

10.7717/peerj.4692/supp-1 Figure S1 Analysis of network topology for various soft-thresholding powers (A) The scale-free fit index (*y*-axis) as a function of the soft-thresholding power (*x*-axis); (B) The mean connectivity (*y*-axis) as a function of the soft-thresholding power (*x*-axis).

10.7717/peerj.4692/supp-2 Table S1 MEs of modules before merging

10.7717/peerj.4692/supp-3 Table S2 MEs of modules after merging

10.7717/peerj.4692/supp-4 Table S3 Genes in each module

10.7717/peerj.4692/supp-5 Table S4 GO enrichment analysis in the CC category

10.7717/peerj.4692/supp-6 Table S5 GO enrichment analysis in the MF category

10.7717/peerj.4692/supp-7 Table S6 GO enrichment analysis in the BP category

10.7717/peerj.4692/supp-8 Table S7 KEGG pathway enrichment analysis

10.7717/peerj.4692/supp-9 Supplemental Information 1 Raw data of TCGA dataset

10.7717/peerj.4692/supp-10 Supplemental Information 2 Raw data of GSE15459 dataset

10.7717/peerj.4692/supp-11 Supplemental Information 3 Raw data of GSE26942 dataset

## Additional Information and Declarations

### Data Availability

The following information was supplied regarding data availability: The raw data has been provided as Supplemental Dataset Files.
