# Supplementary figures and images for: Candidate genes in gastric cancer identified by constructing a weighted gene co-expression network
Source: PeerJ. 2018 May 4;6:e4692. doi: 10.7717/peerj.4692 (PMC5937478; doi:10.7717/peerj.4692)

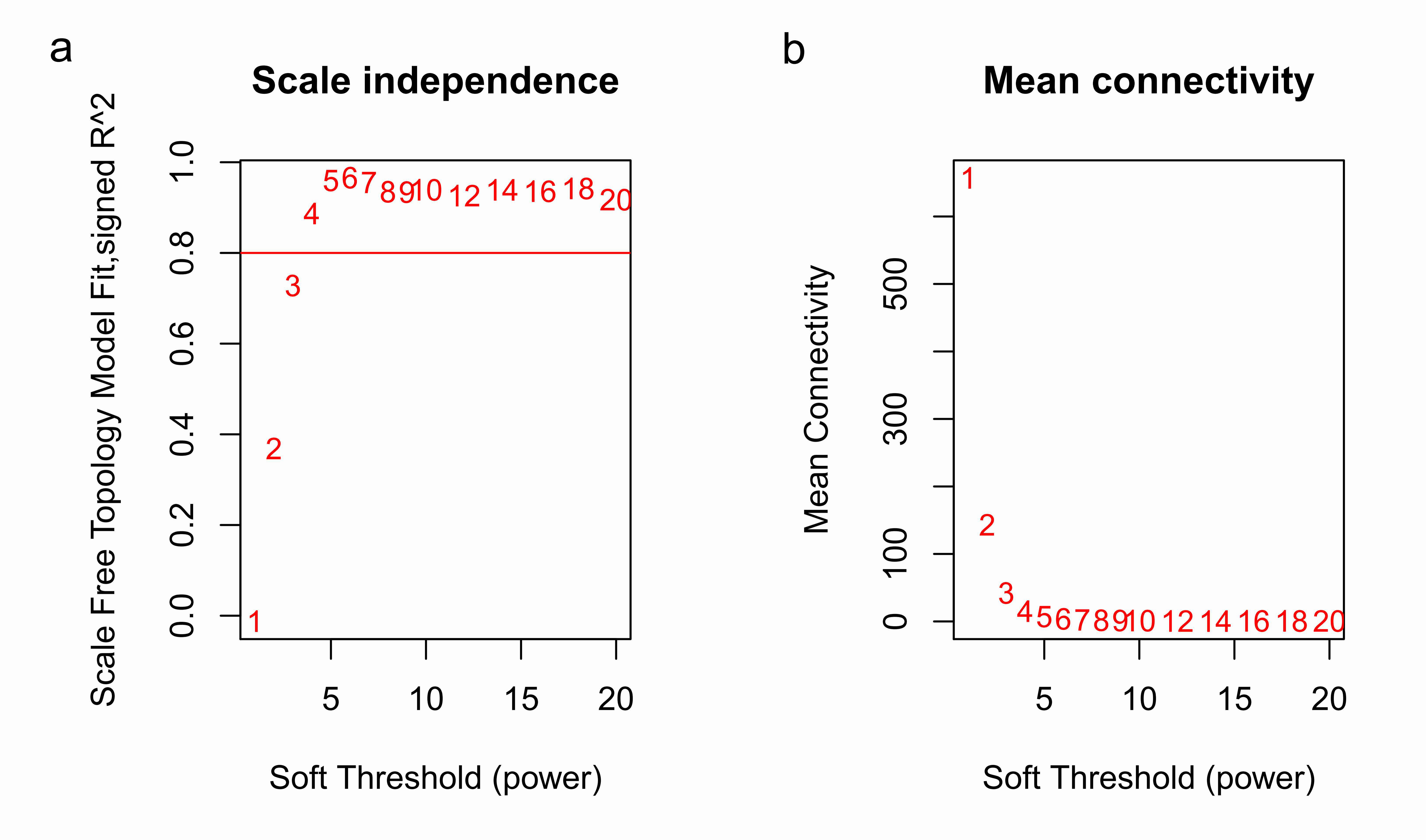

Supplement: Figure S1 — (A) The scale-free fit index (y-axis) as a function of the soft-thresholding power (x-axis); (B) The mean connectivity (y-axis) as a function of the soft-thresholding power (x-axis). [file peerj-06-4692-s001.png]
